# Inbreeding evaluation using microsatellite confirmed inbreeding depression in growth in the *Fenneropenaeus chinensis* natural population

**DOI:** 10.3389/fgene.2023.1077814

**Published:** 2023-02-09

**Authors:** Ding Lyu, Song Sun, Xiujuan Shan, Weiji Wang

**Affiliations:** ^1^ Key Laboratory of Sustainable Development of Marine Fisheries, Ministry of Agriculture and Rural Affairs, Yellow Sea Fisheries Research Institute, Chinese Academy of Fishery Sciences, Qingdao, China; ^2^ Function Laboratory for Marine Fisheries Science and Food Production Processes, Pilot National Laboratory for Marine Science and Technology, Qingdao, China

**Keywords:** inbreeding depression, *Fenneropenaeus chinensis*, microsatellite, body weight, inbreeding coefficient

## Abstract

Understanding inbreeding depressions (IBDs), the effect on the phenotypic performance of inbreeding, is of major importance for evolution and conservation genetics. Inbreeding depressions in aquatic animals were well documented in a domestic or captive population, while there is less evidence of inbreeding depression in natural populations. Chinese shrimp, *Fenneropenaeus chinensis*, is an important species in both aquaculture and fishery activities in China. To investigate inbreeding depression in natural populations, four *Fenneropenaeus chinensis* natural populations (Huanghua, Qinhuangdao, Qingdao, and Haiyang) were collected from the Bohai and Yellow seas. Microsatellite markers were used to evaluate individual inbreeding coefficients (F) of all samples. Furthermore, the effects of inbreeding on growth traits were investigated. The results showed marker-based F was continuous and ranged from 0 to 0.585, with an average of 0.191 ± 0.127, and there was no significant difference among the average F of the four populations. Regression analysis using the four populations showed inbreeding had a very significant (*p* < 0.01) effect on body weight. When analyzing a single population, regression coefficients were also all negative and those in Huanghua and in Qingdao were significant at the level of *p* < 0.05 and < 0.01, respectively. Inbreeding depressions, expressed as the percent change in body weight per 10% increase in F, were 2.75% in Huanghua, 2.22% in Qingdao, and 3.69% in all samples. This study provided a piece of rare evidence of inbreeding depression in natural populations and also guidance toward the conservation of wild *Fenneropenaeus chinensis* resources.

## 1 Introduction

Inbreeding is defined as the mating of individuals that are related by ancestry and results in the reduction of heterozygosity (Falconer and Mackay, 1996). The inbreeding coefficient (F) is a measure of an inbreeding level and can be defined as both the probability that two alleles at any given locus are identical by descent (alleles are descendants from a single ancestor) and the probable proportion of an individual’s loci containing genes that are identical by descent (Falconer and Mackay, 1996; Bourdon, 1997). Inbreeding depression (IBD) is the effect of inbreeding, measured as the reduction in mean phenotypic performance with increasing levels of inbreeding within a population (Falconer and Mackay, 1996; Lynch and Walsh, 1998). The existence of IBD has long been known, especially for fitness traits. Understanding IBD is of major importance for the evolution and conservation of genetics. These effects have been well documented in livestock species (reviewed by Leroy, 2014), and also in aquatic animals (Keys et al., 2004; Zheng et al., 2012; Luo et al., 2014; Gao et al., 2015). However, most of these study examples were carried out in the domestic or captive population, and relatively less evidence of IBD was illustrated in natural populations (Hoffman et al., 2014). The main reason is that F was very accessible in a captive population with pedigree information, in which F of any individual can be obtained by calculating its parents’ coancestry (Falconer and Mackay, 1996). However, investigating the inbreeding level and IBD in natural populations can also be of great significance, and IBD is one of the core research fields in conservation genetics (Frankham et al., 2009). An alternative approach to the pedigree method is calculating F based on molecular markers such as microsatellites (Ritland, 1996; Lynch and Ritland, 1999; Milligan, 2003; Wang, 2007). Compared to the traditional method, F based on a molecular marker can be obtained directly, without the need for pedigree information. As a result, it provided the possibility to study IBD in natural populations.

Chinese shrimp, *F. chinensis*, is an important species in both aquaculture and fishery activities in China. Over the past few decades, the natural population of *F. chinensis* was largely reliant on released shrimps to maintain its size, and their contribution to the total landings has been consistently > 90% (Wang et al., 2006). There is a general belief that a genetic threat of the loss of variation in wild populations was one of the main concerns about an artificial propagation release (Aho et al., 2006; Araki and Schmid, 2010). Also, it was inferred that continuous artificial propagation and release had lowered the level of genetic diversity of *F. chinensis* in Chinese stocks (Wang et al., 2006). Although a previous study demonstrated that inbreeding has a negative effect on economic traits (especially on growth) in the *F. chinensis* breeding population by comparing different levels of inbreeding (Luo et al., 2014), this phenomenon has not been demonstrated under natural conditions. Such a study is important both because inbreeding may affect the extinction risk in wild populations and because understanding IBD is of major importance for the conservation genetics of *F. chinensis*.

In the current study, multiple *F. chinensis* natural populations were collected from the Bohai and Yellow seas in northern China. Microsatellite markers were used to analyze these *F. chinensis* samples to calculate individual F*.* Furthermore, its effects on growth were investigated. The results obtained in this study should provide evidence of IBD in natural populations and also guidance to the conservation of *F. chinensis* resources.

## 2 Materials and methods

### 2.1 Experimental materials

**FIGURE 1:**
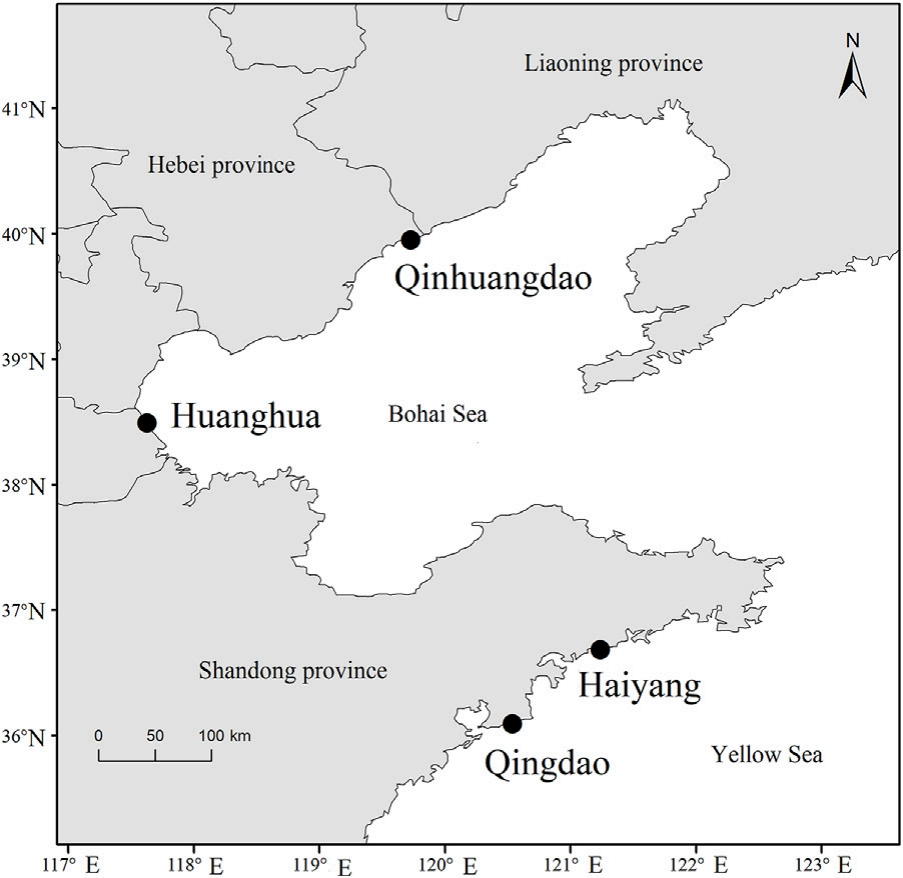
Distribution of *Fenneropenaeus chinensis* sampling locations.

The *F. chinensis* samples were collected from four locations in the Bohai and Yellow seas: Huanghua and Qinhuangdao populations in the Bohai Sea and Qingdao and Haiyang populations in the Yellow Sea (Figure 1). The sampling time in Bohai Sea was in the autumn of 2021 and that in the Yellow Sea was in the spring of 2022. The sample size in each population ranged from 75–194, and the total was 564 (Table 1). All the samples in Qingdao and Haiyang were females because males died after mating in October or November. Those in Huanghua and Qinhuangdao contained both sexes. The body weight of each individual was measured and all samples were transported to the laboratory in liquid nitrogen and stored at −80 °C until analysis.

**TABLE 1:** Sampling locations, time, and number of the four populations.

Location	Sea area	Sampling time	Number
Huanghua	Bohai Sea	11 September 2021	194
Qinhuangdao	Bohai Sea	17 September 2021	136
Qingdao	Yellow Sea	1 to 13 April, 2022	159
Haiyang	Yellow Sea	11 to 19 April, 2022	75

Genomic DNA was extracted from swimming legs in all individuals using standard phenol-chloroform procedures (Sambrook et al., 1989). The primer sequence, fluorescent dyes and anneal of genotyping microsatellite are shown in Table 2. PCR thermal cycling was performed as follows: an initial denaturing at 94°C for 5 min, followed by 30 cycles including 30 s denaturing at 94°C, 30 s annealing at locus-specific temperatures, and 40 s extension at 72°C, and then with a final extension at 72°C for 5 min. The PCR products were separated by an ABI-3130 automated Genetic Analyzer (Applied Biosystems). Alleles from the microsatellite loci were sized with a GeneScanTM-500 LIZ Size Standard (Applied Biosystems) and scored using GeneMapper™ V4.1 (Applied Biosystems).

**TABLE 2:** Primer sequences, fluorescent dyes, and anneal of microsatellites.

Microsatellite	Primer sequence (5′–3′)	Fluorescent dye	Anneal (°C)
EN0033	F: CCT​TGA​CAC​GGC​ATT​GAT​TGG	6-FAM	64
	R: TAC​GTT​GTG​CAA​ACG​CCA​AGC		
RS0622	F: CAG​TCC​GTA​GTT​CAT​ACT​TGG	HEX	66
	R: ACA​TGC​CTT​TGT​GTG​AAA​ACG		
RS1101	F: CGAGTGGCAGCGAGTCCT	ROX	52
	R: TATTCCCACGCTCTTGTC		
RS0683	F: CAC​TCA​CTT​ATG​TCA​CAC​TGC	TAMRA	66
	R: ACA​CAC​CAA​CAC​TCA​ATC​TCC		
EN0113	F: TGT​CAA​GAG​AGC​GAG​AGG​GAG​G	6-FAM	65
	R: TGT​CAA​GAG​AGC​GAG​AGG​GAG​G		
BM29561	F: AAC​AGA​CCA​CAT​ACG​GGA​C	HEX	58
	R: TTT​TCG​GAA​GTA​ACA​TCA​CA		
RS0916	F: GGC​TAA​TGA​TAA​TAA​TGC​TG	ROX	56
	R: CGTTGTTGTTGCTGTTG		
RS0779	F: ATGACACTCAAATCAAAG	TAMRA	50
	R: CAG​AAT​AAC​ATC​ATT​ACT​AC		
FCKR009	F: GCA​CGA​AAA​CAC​ATT​AGT​AGG​A	6-FAM	52
	R: ATA​TCT​GGA​ATG​GCA​AAG​AGT​C		
FCKR002	F: CTC​AAC​CCT​CAC​CTC​AGG​AAC​A	HEX	60
	R: AAT​TGT​GGA​GGC​GAC​TAA​GTT​C		
FCKR0013	F: GCA​CAT​ATA​AGC​ACA​AAC​GCT​C	ROX	61
	R: CTC​TCT​CGC​AAT​CTC​TCC​AAC​T		

### 2.2 Inbreeding coefficient calculation

The genetic diversity parameters, including the number of alleles (N), observed heterozygosity (Ho), expected heterozygosity (He), and polymorphism information content (PIC) at 11 genotyping microsatellite loci were obtained using CERVUS software (Kalinowski et al., 2007). A fixation index ( $F_{st}$ ) was used to measure genetic differentiation among populations and calculated using Arlequin software (Excoffier et al., 2005). There are multiple ways of calculating F from microsatellite genotyping data (Ritland, 1996; Lynch and Ritland, 1999; Milligan, 2003; Wang, 2007), and a triadic likelihood method (Wang, 2007) was adopted in the current study. This was because it was also proven to be the most accurate method to calculate the paired coancestry, having either the lowest root mean error or close to the smallest one in different population structures and sizes, number of loci, and alleles (Wang, 2007; Hammerly et al., 2013). The triadic likelihood method was carried out in coancestry software (Wang, 2011) to obtain F from microsatellite genotyping data.

### 2.3 Inbreeding effect evaluation on body weight

A linear-regression model was used to evaluate the effects of F on body weight:

y=a+b×F+e, (1)

where *y* is the measured value for body weight; *a* is the *y*-intercept; *b* is the regression coefficient of F; and *e* is the residual term. The residual term in this model includes individual genetic effects, day-age effects, and error terms, all of which are unrelated to inbreeding. In particular, since the interaction effect between sex and F was not significant (*p* > 0.05), the analysis of sex effects in Huanghua and Qinhuangdao populations were also subsumed into the residual term.

IBD , expressed as the percent change in phenotype per 10% increase in F, was calculated with the following equation:

$$IBD=\left[\left(b\times 0.1\right)/a\right]\times 100,$$ (2)

where *a* and *b* are *y*-intercept from the regression of *y* on F and the regression coefficient of F, respectively (see Eq. 1). All of the aforementioned statistical analysis processes were performed with corresponding functions in R software (R Development Core Team, 2013).

## 3 Results

### 3.1 Inbreeding coefficient calculation

**FIGURE 2:**
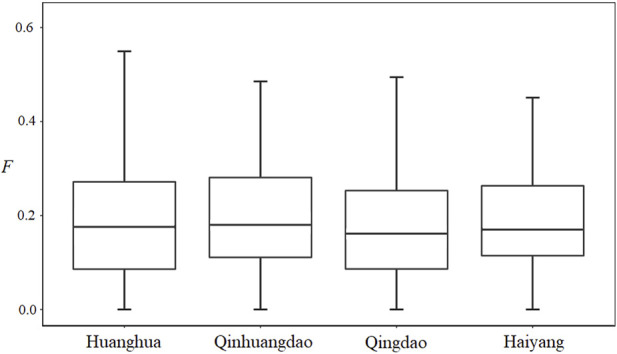
Individual F distribution of *Fenneropenaeus chinensis* in the four populations.

The N, Ho, He, and PIC at each microsatellite locus are shown in Table 3, and the average *N* was 25.18 ± 14.68. The $F_{st}$ ranged from 0.004–0.012 (Table 4), with an average of 0.008 ± 0.003, and only that between Qinhuangdao and Qingdao was significant (*p* < 0.05). The marker-based F was continuous and ranged from 0 to 0.585, with an average of 0.191 ± 0.127. The average F of Qingdao and Haiyang were 0.180 and 0.185, respectively, lower than those of Qinhuangdao and Huanghua, 0.202 and 0.194, respectively (Figure 2). However, there was no significant difference among the average F of the four populations.

**TABLE 3:** Number of alleles (N), observed heterozygosity (Ho), expected heterozygosity (He), and polymorphism Information content (PIC) at 11 microsatellites.

Microsatellite	N	Ho	He	PIC
EN033	55	0.728	0.969	0.966
RS0622	35	0.923	0.954	0.950
RS1101	14	0.762	0.797	0.767
RS0683	38	0.769	0.927	0.920
EN0113	12	0.782	0.862	0.845
BM29561	31	0.898	0.898	0.888
RS0916	4	0.485	0.582	0.490
RS0779	11	0.692	0.872	0.856
FCKR009	28	0.745	0.923	0.916
FCKR002	27	0.825	0.944	0.939
FCKR013	22	0.821	0.920	0.912

**TABLE 4:** Fixation index ( $F_{st}$ ) among geographic populations.

	Huanghua	Qinhuangdao	Qingdao	Haiyang
Huanghua	—	0.141	0.039	0.198
Qinhuangdao	0.007	—	0.093	0.115
Qingdao	0.012*	0.009	—	0.143
Haiyang	0.004	0.008	0.007	—

The lower triangular elements are the fixation index ( $F_{st}$ ) values, and the upper triangular elements are their *p*-values. The asterisk (*) indicates $F_{st}$ is significantly different from zero (*p* < 0.05).

### 3.2 Inbreeding effects on body weight

**FIGURE 3:**
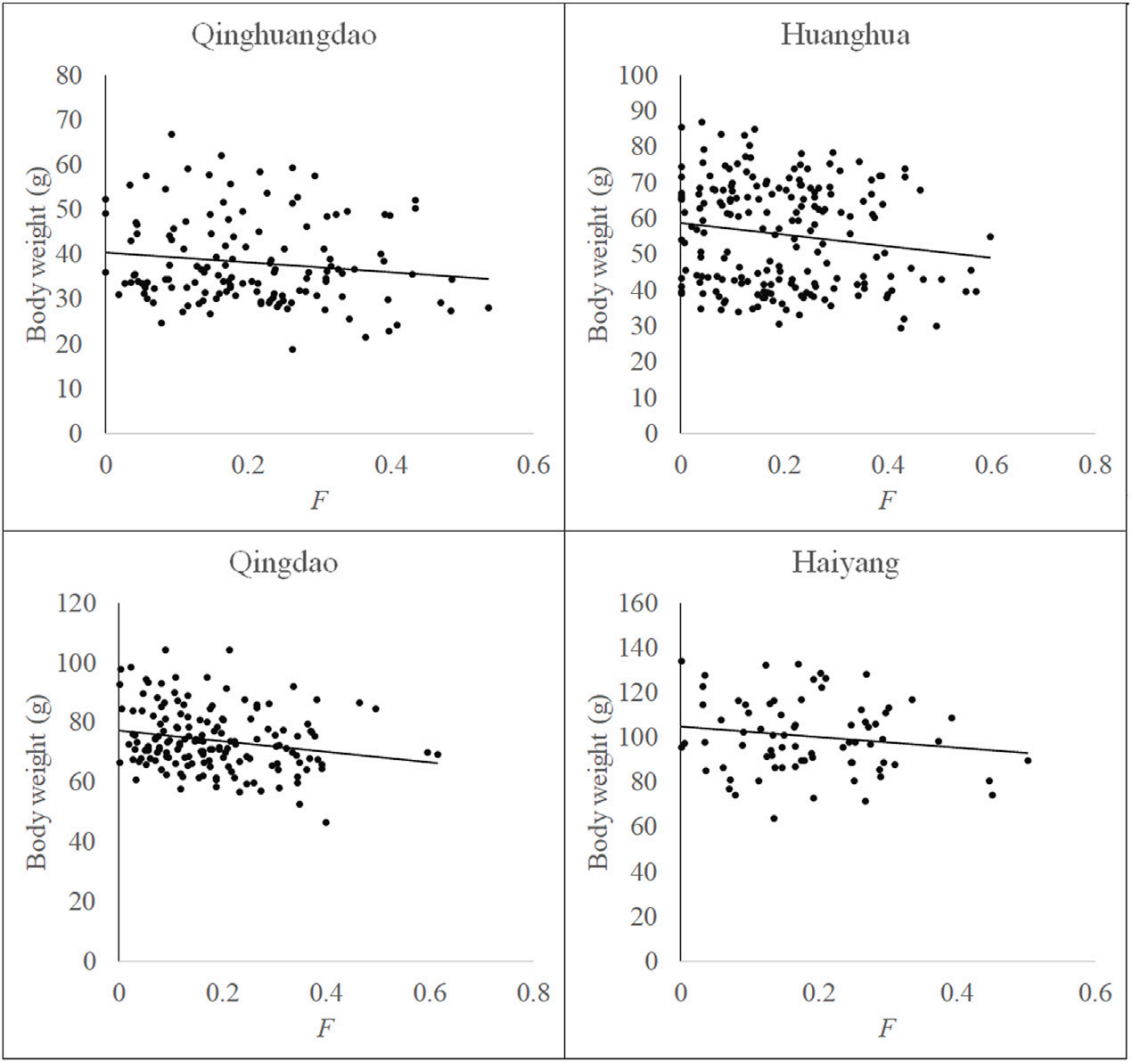
Relationship between F and body weight in each population.

**FIGURE 4:**
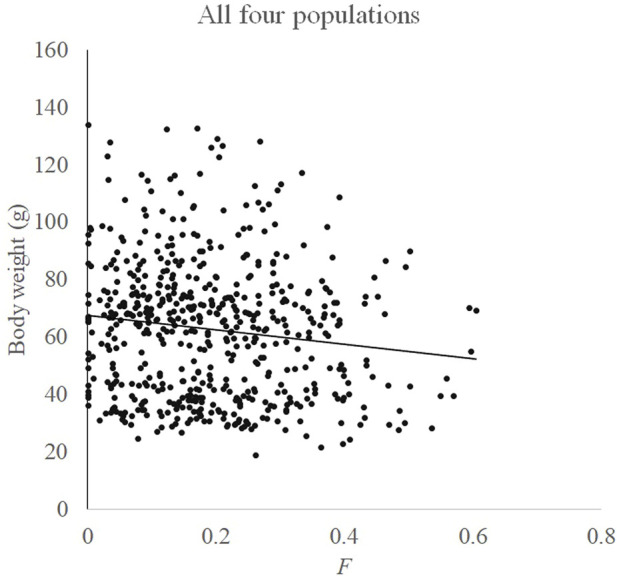
Relationship between F and body weight in all four populations.

Relationships between F and measured body weight in each population and all four populations are shown in Figures 3, 4, respectively. Regression analysis’ results are shown in Table 5. Regression analysis using four populations showed that inbreeding had a very significant (*p* < 0.01) effect on body weight. When analyzing a single population, regression coefficients were also all negative and those in Huanghua and Qingdao were significant at the level of *p* < 0.05 and < 0.01, respectively. However, those in Qinhuangdao and Haiyang were not significantly different from zero due to their high standard errors. In particular, when regression analysis was based on four populations, the interaction effect between the population and F was not significant. For analyses with significant regression coefficients, estimates of IBD on body weight were further calculated. Those in Huanghua and Qingdao were 2.75% and 2.22%, respectively, and when all individuals were fitted, the estimate of IBD was 3.39% (Table 5).

**TABLE 5:** Results from regressions of body weight on F and IBD expressed as percent change in phenotype per 10% increase in F.

Population	*a*	*b*±se	*p*	IBD (%)
Huanghua	58.80	−16.18 ± 7.67	0.036*	−2.75
Qinhuangdao	40.36	−10.86 ± 6.85	0.116	—
Qingdao	77.31	−17.31 ± 6.58	0.009**	−2.22
Haiyang	104.73	−23.70 ± 17.36	0.176	—
All	67.35	−24.70 ± 7.86	0.002**	−3.69

The asterisk (*) indicates *b* is significantly different from zero (*p* < 0.05), and the double asterisk (**) indicates *b* is very significantly different from zero (*p* < 0.01).

## 4 Discussion

We estimated individual F within four *F. chinensis* populations directly, with no requirement of pedigree records or pedigree reconstruction. This method provided an alternative approach to detecting the inbreeding level of *F. chinensis* natural populations. The individual F estimate results showed that there was no significant difference in average F among different populations. All the genetic differentiation between different populations was very low, and only that between Qinhuangdao and Qingdao was significant (*p* < 0.05). This suggested that there was no or very little genetic differentiation among these populations, which was consistent with the expected average F. Most previous studies thought of *F. chinensis* distributed along northern China as one population and suggested a lack of genetic structure in this species (Hwang et al., 1997; Quan et al., 2001; Cui et al., 2007). Furthermore, there was no difference in average F before (Huanghua and Qinhuangdao) and after (Qingdao and Haiyang) the overwintering migration, which suggested that there was no significant relationship between F and the survival rate during overwintering migration. However, the average individual F obtained in this study was consistently high. There are several possible reasons responsible for such a high F. The first is that successive years of artificial propagation and release may have reduced the effective population size of *F. chinensis* and increased the possibility of inbreeding; another possible reason is the occurrence of null alleles that might have also led to Ho in most loci to be lower than that of He.

Based on individual F calculated from microsatellites, we provided a rare piece of evidence of IBD in a natural aquatic animal population as opposed to that in a captive population. The results of the current study showed that F had a significant negative effect on body weight in *F. chinensis* in the wild (*p* < 0.01). In addition, when the samples were divided into four populations according to where they were collected, regression analysis results in two of them supporting this conclusion. Insignificant regression coefficients in another two can likely be attributed to sampling errors which are associated with both a small sample size and a small number of microsatellite loci. Moreover, the interaction effect between the population and F was not significant, which also suggested that IBD should be universal in different populations. Similar to microsatellites, single nucleotide polymorphism (SNP) is another available molecular marker to estimate individual F (Ritland, 1996; Lynch and Ritland, 1999), and F based on whole-genome high-density SNPs should obviously be more accurate. However, the high cost restricts its routine usage in inbreeding level monitoring. If there are no applicable SNP data, using a small number of microsatellites is also an alternative method because microsatellite can provide more marker information contents due to its high polymorphism (Wang, 2016).

Although IBD typically was detected in fitness-related traits (e.g., survival and reproductive traits) (Falconer and Mackay, 1996; Lynch and Walsh, 1998), there was also numerous research on IBD of growth traits in aquaculture species. In another study on *F. chinensis*, Luo et al. (2014) estimated the IBD of body weight as 4.16%–4.74%, which was slightly larger than those in the current study. However, it should be noted that the estimated IBD in Luo et al. (2014) was obtained by comparing several designed high inbreeding levels (*F* = 0.25, 0.375, and 0.50) in a breeding population. Some research has indicated fast inbreeding (mating between close relatives, such as full sibs) caused more harm than slow inbreeding (Day et al., 2003; Thodesen et al., 2005). This view might explain the difference in results between these two studies. In addition, the occurrence of null alleles may affect the accuracy of the estimated F, and the IBD scales were also affected to some extent. Thodesen et al. (2005) summarized previous research on IBD in aquaculture species and found the average IBD of slow inbreeding on growth traits ranging from 0% to 13% with an average of 2.4%, and the results in the current study were fairly consistent with previous research.

In this study, we demonstrated the phenomenon of IBD in a natural population of *F. chinensis* for the first time (to the best of our knowledge). Although a study has demonstrated the existence of IBD in *F. chinensis* breeding population (Luo et al., 2014), the finding in the current study is still of great significance. This is because IBD under laboratory and captive conditions may not be representative of that under natural conditions (Crnokrak and Roff, 1999), and IBD may vary with environmental conditions (Armbruster and Reed, 2005). Over the past few decades, the natural population of *F. chinensis* in China was largely reliant on released shrimps to maintain its size (Deng and Zhuang, 2000; Wang et al., 2006). Due to its strong reproduction ability, often only a few gravid females are needed to produce enough released offspring for a hatchery. Some scholars have long been concerned about the effective population size decreasing in the *F. chinensis* natural population (Wang et al., 2006; Song et al., 2019). Therefore, to avoid IBD, it is very important to maintain the effective population size of the releasing population for *F. chinensis.*

## Data Availability

The original contributions presented in the study are included in the article/supplementary materials, further inquiries can be directed to the corresponding author.
